# Expression of Human NSAID Activated Gene 1 in Mice Leads to Altered Mammary Gland Differentiation and Impaired Lactation

**DOI:** 10.1371/journal.pone.0146518

**Published:** 2016-01-08

**Authors:** April K. Binder, Justin P. Kosak, Kyathanahalli S. Janhardhan, Glenda Moser, Thomas E. Eling, Kenneth S. Korach

**Affiliations:** 1 Reproductive and Developmental Biology Laboratory, National Institute of Environmental Health Sciences, National Institutes of Health, Research Triangle Park, North Carolina, United States of America; 2 Laboratory of Molecular Carcinogenesis, National Institute of Environmental Health Sciences, National Institutes of Health, Research Triangle Park, North Carolina, United States of America; 3 Integrated Laboratory Systems Incorporated, Research Triangle Park, North Carolina, United States of America; University of Rennes-1, FRANCE

## Abstract

Transgenic mice expressing human non-steroidal anti-inflammatory drug activated gene 1 (NAG-1) have less adipose tissue, improved insulin sensitivity, lower insulin levels and are resistant to dietary induced obesity. The hNAG-1 expressing mice are more metabolically active with a higher energy expenditure. This study investigates female reproduction in the hNAG-1 transgenic mice and finds the female mice are fertile but have reduced pup survival after birth. Examination of the mammary glands in these mice suggests that hNAG-1 expressing mice have altered mammary epithelial development during pregnancy, including reduced occupancy of the fat pad and increased apoptosis via TUNEL positive cells on lactation day 2. Pups nursing from hNAG-1 expressing dams have reduced milk spots compared to pups nursing from WT dams. When CD-1 pups were cross-fostered with hNAG-1 or WT dams; reduced milk volume was observed in pups nursing from hNAG-1 dams compared to pups nursing from WT dams in a lactation challenge study. Milk was isolated from WT and hNAG-1 dams, and the milk was found to have secreted NAG-1 protein (approximately 25 ng/mL) from hNAG-1 dams. The WT dams had no detectable hNAG-1 in the milk. A decrease in non-esterified free fatty acids in the milk of hNAG-1 dams was observed. Altered milk composition suggests that the pups were receiving inadequate nutrients during perinatal development. To examine this hypothesis serum was isolated from pups and clinical chemistry points were measured. Male and female pups nursing from hNAG-1 dams had reduced serum triglyceride concentrations. Microarray analysis revealed that genes involved in lipid metabolism are differentially expressed in hNAG-1 mammary glands. Furthermore, the expression of *Cidea*/CIDEA that has been shown to regulate milk lipid secretion in the mammary gland was reduced in hNAG-1 mammary glands. This study suggests that expression of hNAG-1 in mice leads to impaired lactation and reduces pup survival due to altered milk quality and quantity.

## Introduction

Normal female reproduction requires a coordinated effort between hormones and signaling molecules for proper mammary gland development and differentiation during pregnancy and lactation. Mammary gland development occurs during puberty when estrogen levels rise [1] and induces ductal elongation and branching [2, 3]. Further differentiation occurs during pregnancy [4, 5]. At parturition, progesterone levels decrease and prolactin stimulates milk synthesis and secretion [6–8]. The mammary gland responds to a variety of hormones and cell signaling ligands (reviewed in [9]) including members of the Transforming growth factor β (TGFβ) family [10, 11]. TGFβ superfamily members are important for normal reproductive function and successful pregnancy in female mice and are necessary for ovarian development and function [12–14]. TFGβ ligands also regulate mammary gland development, contribute to breast cancer malignancy (reviewed in [11]) and involution of the mammary gland [10]. Nonsteroidal anti-inflammatory drug (NSAID)-activated gene 1 (NAG1), also known as growth differentiation factor 15 (*Gdf15*) and macrophage inhibitory cytokine 1 (*Mic1*) is a member of the TGFβ superfamily [15, 16]. A transgenic mouse that ubiquitously expresses human NAG-1 (hNAG-1) [17] was generated to examine the biological activity of this protein *in vivo*. hNAG-1 mice are leaner than their wild-type (WT) littermates and have reduced white adipose tissue [17] even when maintained on a high-fat diet [18]. Xenografts of hNAG-1 expressing cells into obese C57BL/6 mice reduces adipose tissue with a concomitantly increased expression of several lipolytic and thermogenic genes in both white and brown adipose tissue [18], consistent with increased metabolic activity in hNAG-1 mice. NAG-1 has also been implicated as an appetite suppressor and body weight regulator in mice [19, 20] and cancer patients [21]. In addition to altered metabolism and body weight in hNAG-1 mice [18, 22], the animals have a decreased inflammatory response and are resistant to development of intestinal cancers in both a genetic and chemical induced model [17, 23].

Interestingly, in developing and maintaining hNAG-1 mice it was discovered empirically that hNAG-1 dams crossed to WT males yielded fewer pups at weaning than WT dams crossed with a hNAG-1 male. A decrease was observed in the number of pups weaned from female hNAG-1 expressing mice, while non-transgenic females crossed with males expressing hNAG-1 did not have a significant difference in pups produced [24]. Because in humans circulating levels of NAG-1 are increased in many diseases including cancer, obesity and it is also very high during pregnancy [25] it is important to confirm these preliminary results and then investigate how NAG-1 expression causes the reduction in pups. Herein, we evaluate the consequence of increased expression of hNAG-1 in transgenic mice on female fertility, mammary gland development and lactation, in an effort to better understand the reduced pup survival. Results demonstrate that expression of hNAG-1 reduces the number of pups that survive to weaning, potentially due to reduced lactational sustenance, evidenced by altered mammary gland morphology, the presence of hNAG-1 concentrations (~25ng/mL) in the milk, altered milk composition, smaller milk spots observed in pups and altered gene expression in the lactating mammary gland.

## Materials and Methods

### Animals

All animal procedures were performed under approval from the National Institute of Environmental Health Sciences Institutional Animal Care and Use Committee (Protocol # 06–30 and 01–30) or the Integrated Laboratory Systems Institutional Animal Care and Use Committee. Animals were maintained on a 12h light, 12h dark cycle and fed NIH-31 chow. Transgenic mice were generated to ubiquitously express human NAG-1, and two lines (1377 and 1398) were chosen due to strong expression of the transgene [17]. In addition to genotyping the animals, an ELISA was also performed as previously described to verify increased hNAG-1 in the serum of transgenic animals [23]. Herein, we focus on female hNAG-1 transgenic mice from line 1398, however in some studies animals from both lines were combined for the experiment due to low animal numbers as described in figure legends. Timed pregnant CD-1 foster dams and pups (NIEHS stock) were used to cross-foster as described in figure legends.

#### Fertility study

Our initial breeding studies indicated that hNAG-1 females had fewer pups at weaning than WT females, however it was unknown whether this was due to the number of pups born or survival of the pups. Using a standardized continuous breeding protocol [26], Female mice aged 6 weeks were paired with sexually mature males for the duration of four consecutive litters. This allows the observation of multiple litters in a dam and monitor when pup loss occurred and if the maternal instinct and/or pup survival increased with time. Cages were monitored daily for the presence of copulation plug, the number of litters and pups born. Pups were numbered with toe clippings and the presence of milk spots was qualitatively assessed daily on a scale from 1 to 3, where 1 corresponded to no milk spot and 3 corresponds to a full milk spot visible in the stomach. Pup weight was recorded on post-natal day (PND) 1, 7, 14 and 21 (weaning). Pups were euthanized at weaning (PND21). On PND2, after the fourth litter was born, dams were euthanized, cardiac blood was collected and serum was isolated and frozen at -70°C. Mammary glands (4) were weighed and either snap frozen (right gland, n = 8/group), fixed in 10% formalin (left gland, n = 4/group), or prepared for whole mount histological examination (left gland, n = 4/group) as previously reported [2].

#### Pup serum collection

On PND2, pups were removed from dams and CD-1 pups (n = 5) were cross-fostered with either a WT or hNAG-1 dam to normalize litter sizes and pup genotypes so that transgenic pups were not used. Pups were weighed every 3 days until weaning at PND21 where pups were euthanized and cardiac blood was collected and serum isolated and stored at -70°C until further analysis.

#### Lactation challenge

On PND1 all the pups were removed from dams and CD-1 pups from timed pregnant CD-1 dams (n = 8) were placed with either a WT or hNAG-1 dam to normalize the litter sizes and pup genotypes. The morning of PND2 or PND9 pups were separated from dams for 3 hours. Pups were weighed and then the dam was added back to the home cage and monitored. Once suckling had begun (evidenced by hunched posture with several pups attached) the dam was allowed to nurse for 30 minutes at which point the pups were sexed, weighed and returned to foster dam (PND2) or euthanized (PND9). Blood was collected and serum isolated from pups on PND9, serum was combined from 2 animals of the same sex and stored at -70°C until further analysis.

#### Milk collection

On PND1 pups were removed and CD-1 pups (n = 5) were placed with WT or hNAG-1 dams to normalize litter sizes. On PND2, milk was collected following a modified protocol similar to that reported by Fenton et al [27, 28]. Briefly, dams were separated from pups for 3 hours and then given oxytocin (2U/mL, IP injection) 10 minutes before manual manipulation of the nipples and milk was collected using a sterile pipette. Milk samples were stored at -70°C until further analysis. Dams and mouse pups were euthanized.

### Clinical Chemistry Analysis

Serum and milk samples were stored at -70°C until analyses were done. Total Triglycerides and Cholesterol kits were purchased from Beckman Coulter (Melvill, NY) and the non-esterified fatty acid (NEFA) kit was purchased from Sekisui Biagnostics (Exton, PA) according to manufacturer’s instructions. Samples were run in duplicate and analysis was performed using an Olympus AU400e (Beckman Coulter, Inc., Irving, TX).

### Histology

Formalin-fixed, paraffin-embedded mouse mammary glands (PND2 glands collected from fertility study) were sectioned and stained using standard H&E staining protocol. TUNEL (Terminal deoxynucleotidyl transferase dUTP nick end labeling) immunohistochemical analysis was done to detect apoptosis. Formalin -fixed, paraffin-embedded mouse mammary glands (PND2 glands collected from fertility study) were stained with ApopTag Plus Peroxidase *In Situ* Apoptosis Detection Kit (Cat# S7101, Millipore, Billerica, MA) using the manufacturer’s recommendations. Staining was visualized using 3-diaminobenzidine (DAB) chromagen (DakoCytomation, Carpenteria, CA) and counterstained with hematoxylin. The slides were dehydrated through graded ethanol, cleared in xylene, and coverslipped.

### Gene Expression and Protein Analysis

Mammary gland number 4 was collected from dams on PND2 after their 4^th^ litter and snap frozen from animals used in the fertility study. The glands were pulverized individually and RNA was isolated using TRIzol (Invitrogen, Carlsbad, CA) following the manufacturer’s instructions. RNA concentration and quality was determined by spectrophotometry and cDNA was reverse transcribed using the SuperScript First-Strand Synthesis System (Invitrogen, Carlsbad, CA). Primers used for *Cidea* were forward 5’-TGACATTCATGGGATTGCAGAC-3’ and reverse 5’-GGCCAGTTGTGATGACTAAGAC-3’ [29] and Cyclophilin B (officially *Ppib*) were forward 5′-CAAAGACACCAATGGCTCACAG-3′ and reverse 5′-CCACATCCATGCCCTCTAGAAC-3′ [30]. Data are shown as a ratio of *Cidea/cyclophilin B* as described previously [31]. Total protein homogenates were prepared using a fraction of the pulverized glands, and 10 ug of protein was used for Western blot analysis as previously described [30]. Briefly 10 μg protein was run on polyacrylamide SDS gel and transferred to a nitrocellulose membrane. Membranes were blocked in 5% nonfat milk in Tris-buffered saline containing 0.05% Tween (TBST) and then incubated overnight with anti-CIDEA antibody (#ab8402, Abcam, Cambrindge, MA) or anti-beta-actin antibody (#3700, Cell Signaling, Beverly, MA) diluted in 5% milk in TBST. Membranes were rinsed and incubated in horseradish peroxidase-conjugated secondary antibody (Cell Signaling, Beverly, MA). The membrane was then rinsed and antigen-antibody complexes were detected by Chemiluminescence using Pierce ECL Plus (Thermo Scientific, Rockford, IL) following manufactures protocol.

#### Microarray analysis

Mammary gland number 4 was collected from dams on lactation day 2 (L2) and RNA was isolated as described above in gene expression and protein analysis subsection. Gene expression analysis was performed in the NIEHS Microarray Core using Agilent Whole Mouse Genome 4x44 format oligo arrays (014868) following manufactures protocol. Five hundred ng RNA was Cy3-labeled cRNA from WT (n = 4) or hNAG-1 expressing (n = 5) expressing mice. The Cy3 labeled cRNAs were fragmented and hybridized for 17 hours in rotating hybridization oven, washed and scanned with an Agilent Scanner. Data was obtained using Agilent Feature Extraction software (v9.5) and data was analyzed using OmicSoft Array Studio software (v7.0). Differentially expressed probes were identified using ANOVA and a p-value of p<0.05 significant differences between transcripts in WT or hNAG-1 expressing mice. In addition, a fold change of 1.5 and signal intensity of at least 65 in 1 sample were used. Pathway analysis was performed using Ingenuity Pathway Analysis (IPA; Ingenuity Systems Inc, Redwood City, CA). All microarray data files are available from the GEO database (accession # GSE71125).

## Results

### Expression of hNAG-1 in female mice causes reduced pup survival and impaired lactation

Female transgenic mice expressing hNAG-1 were set up in a continuous breeding study to evaluate reduced fertility. Male hNAG-1 expressing mice do not have observable defects in fertility [24] and transmission of hNAG-1 gene through males shows no difference in pups. Female hNAG-1 expressing mice took slightly, albeit significantly, longer time to become pregnant compared to their WT littermates (Table 1). Examination of the estrous cycle in WT vs. hNAG-1 mice revealed that the hNAG-1 females have a slightly longer cycle than normal (WT 4.7±0.1 days vs. hNAG-1 5.4±0.3 days, p = 0.07). Transgenic animals are able to carry the pups to term, however the number of pups that survived to weaning age (PND21) was significantly lower in hNAG-1 mice compared to WT mice (Table 1), suggesting that hNAG-1 dams were not able to care for or sustain the nutritional needs of their pups as well as WT dams.

**Table 1:** Continuous breeding study of hNAG-1 transgenic females paired with proven male for 4 litters.

Genotype	Age at first mating (weeks)	Age at first pregnancy (weeks)	Pups Born (Ave/litter)	Pups Weaned (Ave/litter)	Relative Mammary Gland Weight (g)
WT (n = 8)	6.12 ± 0.05	7.14 ± 0.62	7.31 ± 0.66	3.56 ± 0.56	1.90±0.18
hNAG-1 (n = 8)	6.09 ± 0.05	8.27 ± 0.79*	6.19 ± 0.50	1.38 ± 0.40**	1.13±0.11 **

Data shown is average +/- SEM and statistics were analyzed by Mann-Whitney tests. P values are two sided for age at mating and first successful pregnancy and one sided for number of pups born and weaned compared to WT for each line.

* p<0.05

** p<0.01

**Fig 1:**
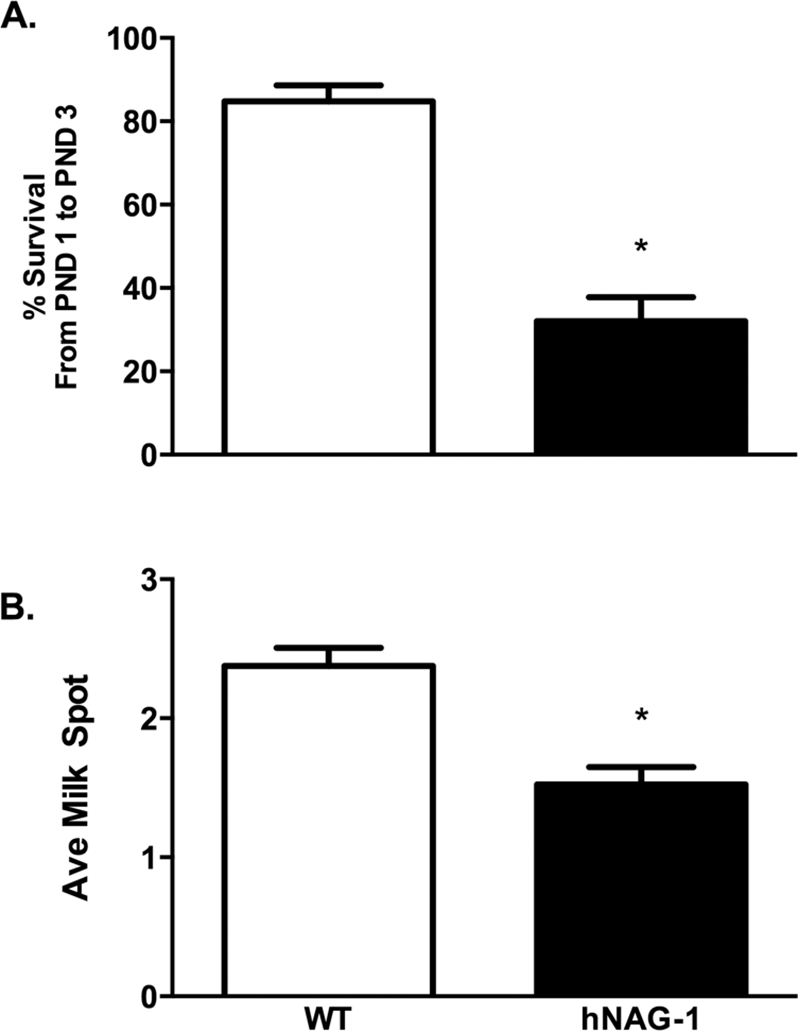
Reduced survival and milk spots early in lactation. A.) Percent pup survival to PND3 in pups born to WT and hNAG-1 dams. The data shown is average percent survival +/- SEM across 4 litters. B.) Qualitative analysis of milk spots scored on the day of birth through day PND2 and then averaged for each dam/litter. Data shown is average over 4 litters +/- SEM. Mann-Whitney statistical test was used to compare hNAG-1 transgenic dams to WT control. *, p<0.05.

Many pups were lost in the first few days after birth, and those that were able to survive longer than 72 hours typically survived to weaning. Pup survival to PND3 is reduced by 53% in hNAG-1 mice compared to WT mice (p<0.05) across 4 litters (Fig 1A). Qualitative analysis of milk spots was performed [1 = no spot, 2 = partial spot, 3 = full spot] and pups born to WT dams had an average milk spot score of 2.4 compared to 1.5 (p<0.05) for pups nursing from hNAG-1 dams (Fig 1B). The altered pup survival and smaller milk spots observed suggests that the expression of hNAG-1 in the mouse may affect mammary gland development, function and/or lactation.

### Mammary gland defects in hNAG-1 expressing mice

Whole mount analysis of the number 4 mammary glands demonstrate that ductal growth, elongation and branching is not altered in virgin glands (S1 Fig), although hNAG-1 virgin mammary glands are smaller even when corrected for body weight (5.7±0.9 mg/g (hNAG-1) compared to 7.9±1.4 mg/g (WT)). Whole mount analysis of the number 4 gland is not different in virgin adult mice (10–12 weeks), the glands have varied development in dams on lactation day 2 (L2). While some of the hNAG-1 females have glands that appear similar to WT females, several hNAG-1 glands appear to be immature or undergoing involution (S1 Fig). The glands with the immature morphology are the same dams that were unable to maintain pup survival during the fertility study. The average relative mammary gland weight from lactating dams is reduced in hNAG-1 mice compared to WT mice (Table 1).

**Fig 2:**
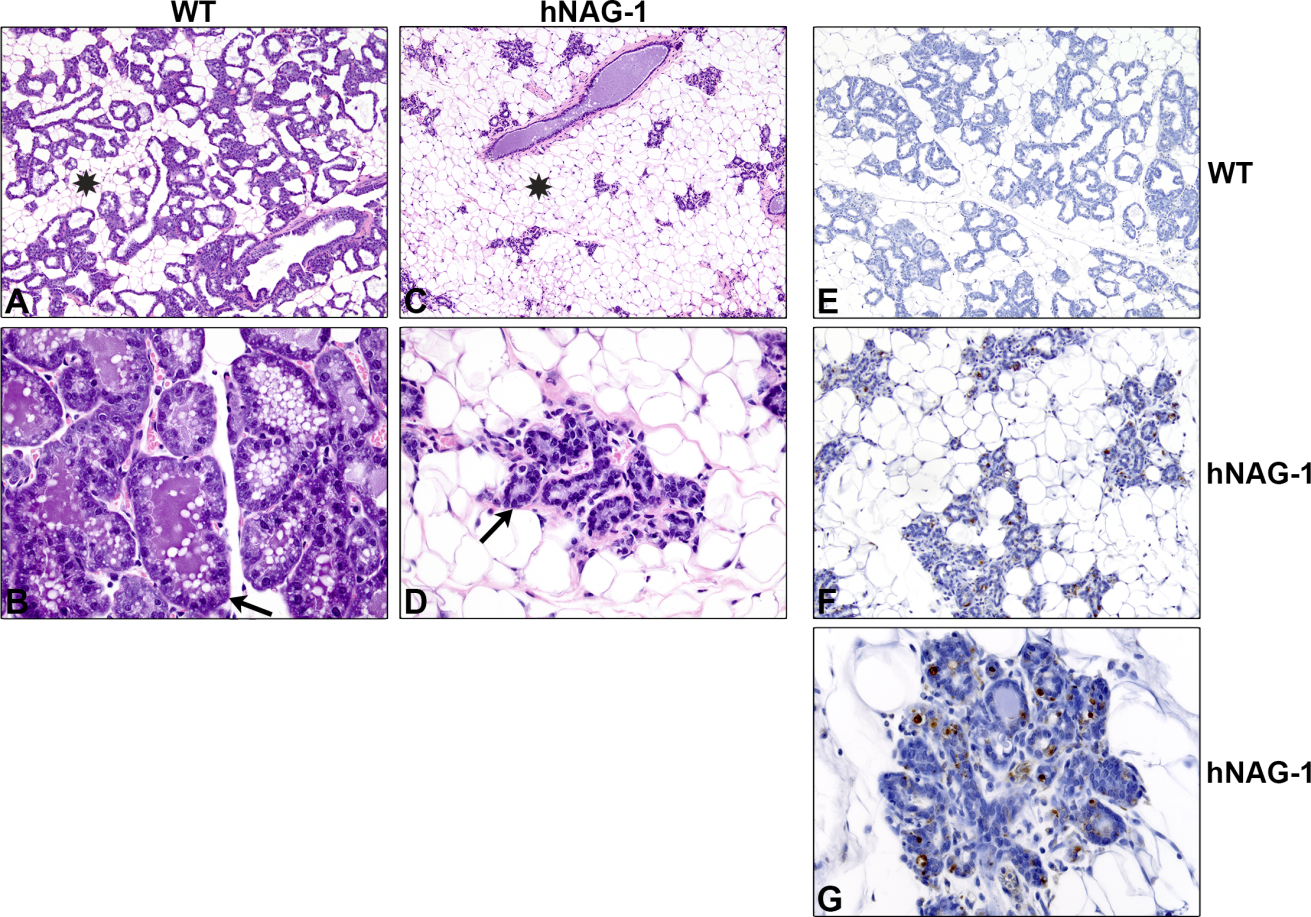
Histological examination of mammary glands from WT and hNAG-1 mice. Hematoxylin and eosin staining of WT and hNAG-1 mammary glands on L2 show that hNAG-1 (C&D) mice have reduced occupancy of the fat pad by mammary gland compared to WT (A&B) as denoted by the asterisk. Higher magnification shows that the acini in WT mammary glands (B) are lined with plump cuboidal cells while the hNAG-1 mammary glands (D) have low cuboidal cells and contain only small amount of secretion in their lumen (arrows). TUNEL staining (E-G) was done on L2 WT (E) and hNAG-1 (F&G) mammary glands. Representative images show DAB chromogen with hematoxylin counterstain.

Histological analysis of the mammary glands from lactation day 2, demonstrates that in the WT mammary gland acini and ducts occupy the majority of the fat pad, whereas in the hNAG-1 mammary gland only a small percentage of the fat pad is occupied by epithelium (Fig 2A–2D). Higher magnification demonstrates that in the WT mice (Fig 2B) plump cuboidal cells line the acini and the lumens contain clear lipid vacuoles. In the most affected hNAG-1 mice, the acini are lined by flat epithelium and associated with very minimal or no secretion in the lumen (Fig 2D). The morphology of the glands in the whole mount analysis suggests that some of the glands appear to be undergoing early involution, potentially due to increased apoptosis. TUNEL staining demonstrates that hNAG-1 expressing mice have increased TUNEL positive cells on L2 compared to WT mammary glands (Fig 2E–2G).

**Fig 3:**
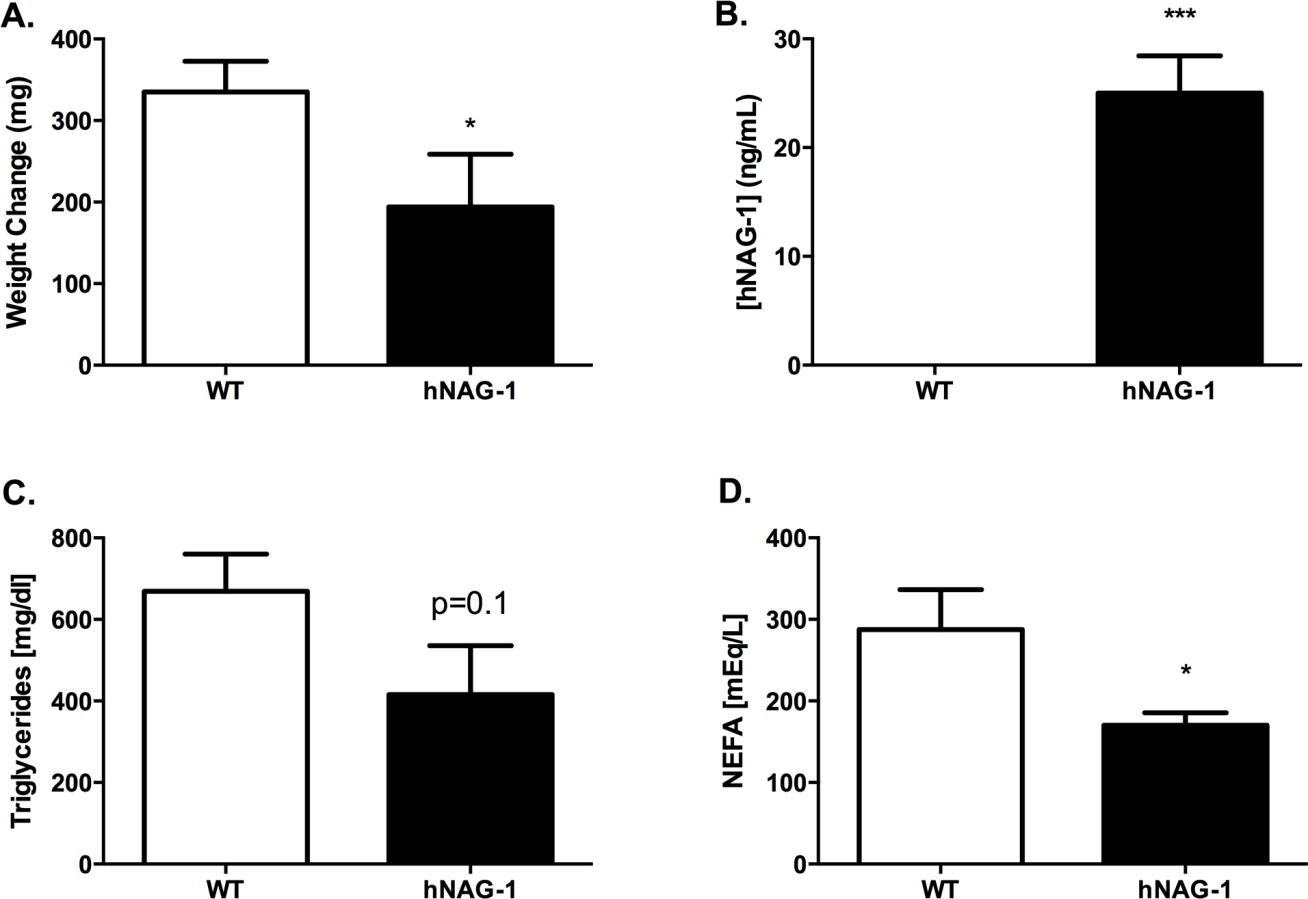
Reduced pup growth and altered milk composition in hNAG-1 dams. A.) On L2, Eight CD-1 pups were separated from their dam for 3 hours, weighed and placed back with the dam. Pups were allowed to nurse for 30 minutes and weighed again. Weight change due to increased milk intake was calculated. B-D.) Milk was isolated from WT (n = 4) and hNAG-1 (n = 6) mice on lactation day 2. ELISA to look at hNAG-1 concentrations present in milk (A), total triglycerides (B) or NEFA (C) in milk isolated from WT or hNAG-1 transgenic dams. Data shown is average +/- SEM. Mann-Whitney statistical test was used to compare hNAG-1 transgenic to WT dams. *, p<0.05 and ***, p<0.001.

The reduced milk spots on the pups nursing from hNAG-1 transgenic dams suggest that lactation may be altered in hNAG-1 dams compared to WT dams. To evaluate milk quantity, a lactation challenge was performed on L2. Briefly, 8 CD-1 pups were placed overnight with dams on lactation day 1 and then separated on L2 for 3 hours. CD-1 pups were used to eliminate the possibility that pups carrying the hNAG-1 transgene may confound the phenotype and to eliminate the thriftiness of the pups from affecting nursing ability. CD-1 pups were randomly assigned to either an hNAG-1 or WT dam. Following 30 minutes of nursing, the change in pup weight was used as a measure of milk volume. The weight change in pups nursing WT dams was 335±38 mg compared to 194±65 mg in hNAG-1 dams (p<0.05) (Fig 3A) demonstrating reduced milk volume in pups nursing from transgenic hNAG-1 expressing mice.

### Alterations in pup growth and milk composition in hNAG-1 mice

Altered milk quantity and quality may both directly contribute to the reduced pup survival. To examine milk composition, milk was collected from WT and hNAG-1 dams on L2. Measureable hNAG-1 (approximately 25 ng/mL) was present in milk isolated from hNAG-1 dams while WT dams had undetectable levels (Fig 3B). Furthermore, there was a decrease in total triglycerides in the milk collected from hNAG-1 dams compared to WT dams (2,079±599 vs. 3,344±374 mg/dl, p = 0.1) although this difference was not statistically significant (Fig 3C). Milk secreted from hNAG-1 dams had significantly reduced non-esterified free fatty acids (NEFA) levels compared to WT dams (8,500±783 vs. 14,375±1994 mEq/L, p<0.05) (Fig 3D).

**Fig 4:**
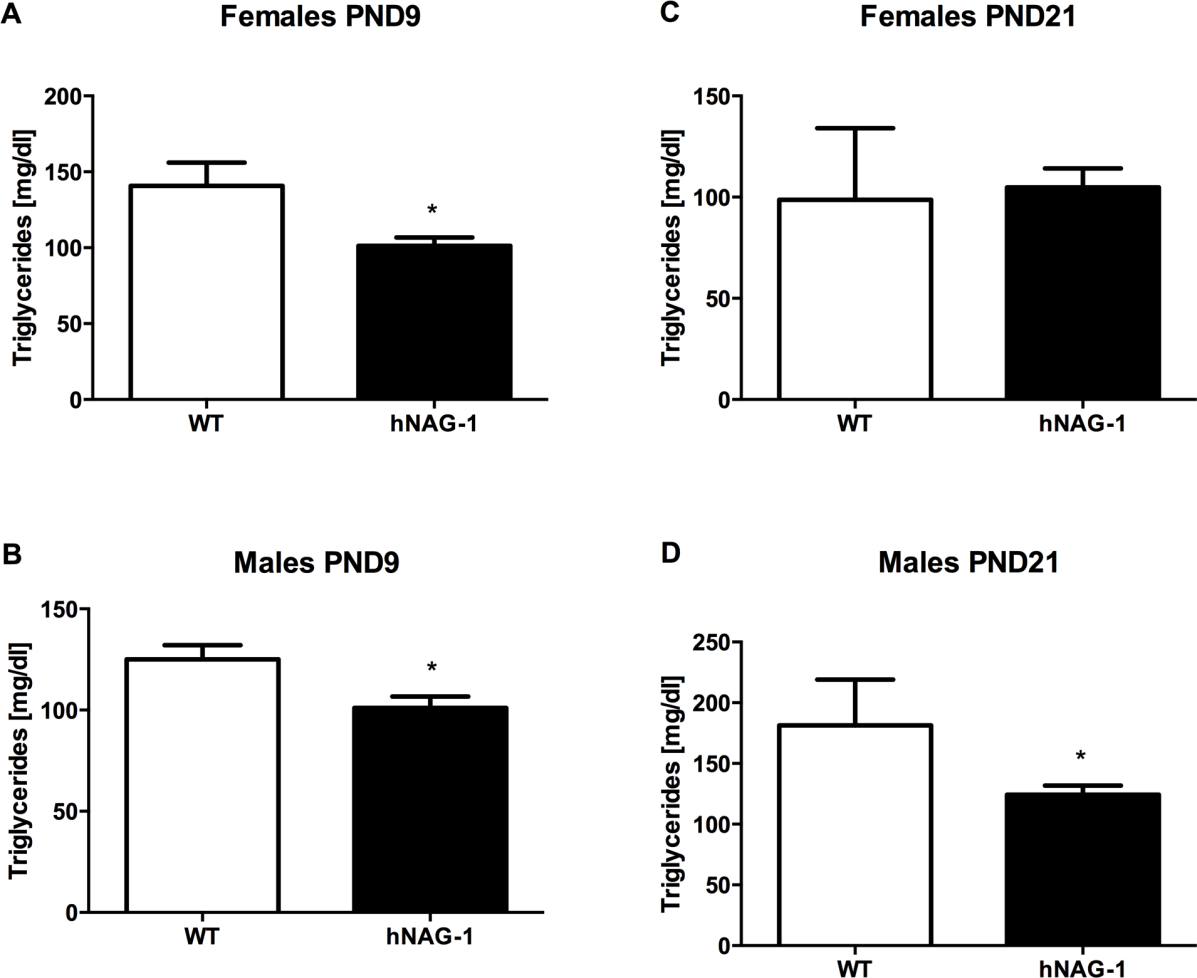
Pup serum shows reduced triglycerides during mid-lactation (PND9) and at weaning (PND21) from hNAG-1 dams compared to WT dams. CD-1 pups were cross-fostered with WT or hNAG-1 dams on PND2 so the pups are all WT CD-1 pups. Pup serum was examined for Triglycerides at PND 9 (A&B) or PND21 (C&D). Data shown is average +/- SEM. Mann-Whitney statistical test was done to compare each line to WT. *, p<0.05.

To evaluate whether pups are receiving proper nutrition while nursing, serum was collected from CD-1 pups cross-fostered with WT or hNAG-1 transgenic dams at PND9 (nursing diet) and PND21 (nursing and chow diet) and NEFA, cholesterol and triglyceride concentrations were measured. Total serum triglycerides are reduced in pups nursing from hNAG-1 dams, with reduction more significant in male pups than female pups. Female pups nursing from hNAG-1 dams had serum triglyceride 39 mg/dl lower than WT (101 vs. 140 mg/dl, p<0.05) (Fig 4A), while male pups had a reduction of 24 mg/dl (101 vs. 125 mg/dl, p<0.01) (Fig 4B). A reduction in triglyceride concentration was also observed in male pups at weaning age (PND21) from pups cross-fostered with hNAG-1 dams compared to WT dams. Female pups did not show a significant difference in triglyceride concentrations, possibly due to the deviation and small sample size (Fig 4C). Male pups with hNAG-1 dams had reduced serum triglyceride concentrations, with hNAG-1 dams 57 mg/dl lower than WT (124 vs. 181 mg/dl, p<0.05) (Fig 4D). Cholesterol concentrations (S2 Fig) and the concentrations of NEFA (S3 Fig) in pup serum from both lines of mice are similar to those in pups nursing from WT dams.

### Altered gene expression in mammary glands of hNAG-1 mice

A microarray analysis was performed to examine for differences of gene expression in the mammary gland tissue isolated from WT or hNAG-1 dams on L2. PCA analysis of the data demonstrates that while there is some variability, the samples form two distinct groups based on genotypes. Initial analysis shows 2,386 probes are differentially expressed between WT and hNAG-1 mice, however setting a more stringent analysis using fold change cutoff of 1.5 and intensity signal of at least 65 in one sample reduced the number of probes to 289. Of these significant probes, 248 mapped to known genes by Ingenuity Pathway Analysis (IPA) and 41 were unmapped. Interestingly, the top 20 genes that show either increased or decreased expression have not been reported previously to function in mammary gland development. Pathway analysis in IPA demonstrated that the top molecular and cellular functions include those involved in cellular signaling, lipid metabolism and small molecule biochemistry as listed in Table 2. The altered expression of genes within these networks may contribute to the impaired lactation observed in hNAG-1 females.

**Table 2:** Top Molecular and Cellular Functions.

Molecular and Cellular Functions	p-value	# molecules
Cell Signaling	6.47E-07–2.48E-02	17
Lipid Metabolism	1.55E-04–2.48E-02	22
Molecular Transport	1.55E-04–2.31E-02	12
Small Molecule Biochemistry	1.55E-04–2.48E-02	36
Cellular Compromise	2.16E-04–2.48E-02	8

**Fig 5:**
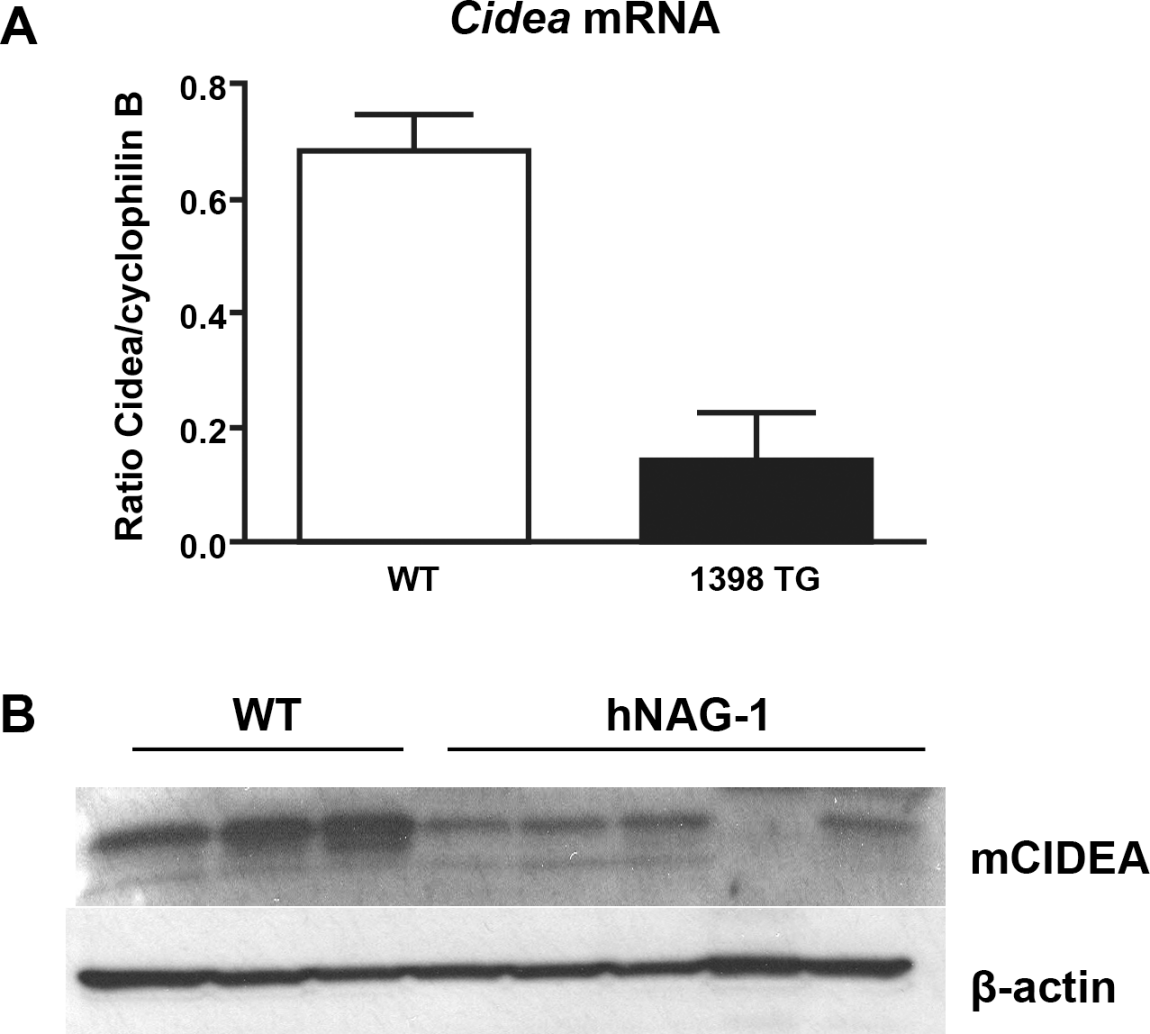
hNAG-1 mammary glands have reduced C*idea*/CIDEA expression. Mammary glands were isolated on L2 and RNA or protein was isolated from WT and hNAG-1 mice. A.) Quantitative real-time PCR was performed using primers specific for *Cidea* and data was normalized to cyclophilin B (*Pip1b*). Data shown is average +/- SEM. Mann-Whitney statistical test was used to compare each line to WT control. ***, p<0.001. B.) Whole cell lysates were run on a SDS polyacrylamide gel and then immunoblotted using antibodies specific for CIDEA and beta-actin, used as a loading control. Data shown are from independent animals, WT (n = 3) and hNAG-1 (n = 5).

*Cidea*, a transcriptional co-activator of C/EBPβ in mammary glands, has been shown to regulate the secretion of milk lipids from mammary glands of mice. Pups born to dams lacking the co-activator *Cidea* have reduced pup survival to PND3 with impaired milk lipid concentrations observed in Cidea^-/-^ dams compared to Cidea^+/-^ dams [29]. Cidea^-/-^ mice are lean with reduced white adipose tissue and are resistant to obesity on a high fat diet for an extended time [32] similar to observations made in adult male and female hNAG-1 transgenic mice [18]. Cidea^-/-^ mice have similar lactation deficiencies [29] as observed in hNAG-1 expressing mice. From these results we propose the hypothesis that the expression of *Cidea* in the mammary gland will be lower in the NAG-1 mice than the WT mice. To obtain data in support this hypothesis, we measured *Cidea* expression in the mammary gland of hNAG-1 mice on L2. *Cidea*/CIDEA expression was measured by mRNA levels and CIDEA protein levels are reduced in the mammary gland tissue from hNAG-1 mice in both lines (Fig 5).

The expression of hNAG-1 in female mice does not affect reproduction or fertility, but diminishes the amount and quality of secreted milk from the mammary gland and thus reduces pup survival. These effects in female hNAG-1 mice appears to be related to the changes in mammary gland, which may alter metabolic activity and suppress expression of *Cidea*/CIDEA, known to regulate the secretion of milk lipids [29].

## Discussion

The expression of hNAG-1 in mice has previously been shown to lead to reduced body weight, and white adipose tissue mass in animals on a high fat diet [17] and are resistant to obesity, presumably due, in part, to increased metabolic activity and energy expenditure [18]. In the current study, we demonstrate that hNAG-1 expression contributes to altered mammary gland morphology and function, leading to a reduction in milk quantity and quality. Milk spot analysis and lactation challenges show that hNAG-1 dams produce less milk than WT dams. Milk composition in hNAG-1 dams is also altered, including the presence of mature hNAG-1 protein and reduced NEFA concentrations in the secreted milk. These differences may contribute to lowered pup serum triglyceride levels and significantly reduced pup survival.

The mammary gland goes through multiple stages of development during pregnancy and lactation culminating with involution of the gland and return to its pre-pregnancy state [9]. Interestingly, TGFβ ligands can act to suppress lactation and regulate involution [10]. Previous studies have shown that NAG-1, a TGFβ superfamily member, can increase apoptosis in *in vivo* cultured ovarian cancer cells [33], and in our current study we show that mammary glands from hNAG-1 dams have increased TUNEL positive cells compared to WT mammary glands. The mammary glands from hNAG-1 dams also have reduced occupancy of the fat pads compared to WT dams, with flattened cells lining the acini and only a small amount of lipid secretion in the lumen (Fig 2). Pups born to hNAG-1 females have reduced survival to PND3 and reduced milk spots compared to those born to WT females (Fig 1) suggesting that hNAG-1 dams are not able to provide the nutritional support for their pups as well as WT dams, which is consistent with the morphological deficiencies seen in several hNAG-1 mammary glands. Importantly, the reduced survival was not due to the presence of the transgene in the pups as demonstrated by cross-foster pilot experiments and pup survival when the transgene comes from male hNAG-1 mice crossed with WT females.

The majority of pups lost to hNAG-1 mice occurred in the first 72 hours after birth, suggesting reduced thriftiness and fitness of pups born to hNAG-1 mice. Cross-fostering of CD-1 pups with hNAG-1 dams on L2 did not affect CD-1 pup survival, however these CD-1 pups are larger than those born to C57BL/6 females and were well fed immediately after birth by their CD-1 mother. Even though reduced pup survival was only present in C57BL/6 pups, reduced weight gain during early development was observed in both C57BL/6 pups and CD-1 pups nursing from hNAG-1 dams compared to those nursing from WT dams. Reduced milk accumulation was also observed in CD-1 pups cross-fostered with hNAG-1 dams (Fig 3A), even though the CD-1 pups were consistently attached and suckling suggesting that while pup aggressiveness or thriftiness may partially explain the phenotype, reduced secretion from the hNAG-1 dam glands is the most likely contributor.

Malnutrition in early life is a major public health concern in low income regions that contributes to increased mortality and disease states later in life [34]. In rats, malnutrition during lactation contributes to an altered metabolic state where the offspring developed diabetes and became insulin resistant [35, 36]. Mice lacking the PPARγ response element in the *Pck1* promoter had reduced milk triglycerides that led to altered metabolic function of offspring nursing from these dams later in life [37]. Growth and development of perinatal mice requires a large source of energy, which in the mouse is primarily obtained from triglycerides. While milk triglycerides were not significantly different between WT and hNAG-1 dams, there was a reduction of serum triglycerides in pups nursing from hNAG-1 dams. These findings are similar to that observed in hNAG-1 adult mice [18], which have reduced serum triglyceride levels while FFA levels remain unchanged. This reduction could also be due to the reduced milk consumption observed in pups nursing from hNAG-1 dams. There are several knockout mouse models that show reduced milk triglyceride levels, including mice lacking the transcriptional co-activator *Cidea* [29], glycerolipid acyltransferase 6 (*Agpat6*) [38] or Diacylglycerol acyltransferase 1 (*Dgat1*) [39]. The pups born to dams from these knockout mouse models have reduced survival and or weight gain during early development, which suggests overlapping pathways or function. In addition to reduced milk triglyceride concentrations, these mice also have reduced body weights compared to their WT littermates similar to hNAG-1 adult mice. Transgenic mice overexpressing human lipoprotein lipase in the mammary gland also show reduced milk triglyceride levels, which contribute to a growth delay and reduced serum triglyceride levels in the pups fed transgenic milk [40].

Cidea^-/-^ mice have reduced lipids in their milk compared to Cidea^+/-^ mice and contribute to the reduced pup survival observed in these mice [29]. Cholesterol levels were slightly reduced on PND10, while FFA levels in the pups serum remained unchanged in the pups nursing from the hLPL transgenic mice [40], similar to findings in the hNAG-1 fed pups. C*idea*/CIDEA expression levels were also reduced (Fig 5) in the hNAG-1 mammary gland suggesting that hNAG-1 may indirectly regulate *Cidea* expression. While the microarray analysis did not show differences in *Cidea* expression, there were several genes implicated in lipid metabolism that are differentially expressed between WT and hNAG-1 mammary glands. Lipid synthesis is important in the mammary gland to provide proper milk composition, and altered expression of these genes may contribute to reduced nutrition of suckling pups.

The overall lipid profile of milk from hNAG-1 mice was not different from WT littermates, yet a relatively high level of hNAG-1 was present in the milk from transgenic dams (~25 ng/mL, Fig 5A). The circulating level of hNAG-1 in adult transgenic mice is approximately 50 ng/mL [17], demonstrating that the pups are ingesting a high concentration of hNAG-1, especially when corrected for size. Secreted hNAG-1 was not observed in the serum isolated from nursing pups, suggesting that the pups metabolized the hNAG-1. Adult hNAG-1 mice have reduced white adipose tissue and are resistant to obesity, show reduced inflammatory responses and increased insulin sensitivity, presumably due to increased thermogenesis and metabolic activity [17, 18, 22, 23] in the presence of circulating hNAG-1. Furthermore, this altered nutritional state during the important perinatal development period in pups may contribute to altered metabolic properties in adulthood, which remains to be studied.

In our current study, expression of hNAG-1 protein in female mice causes altered reproductive capacity, by causing reduced pup growth and survival. The mammary glands of hNAG-1 dams showed altered morphology and produce reduced quantities of milk, even when pups are actively suckling. The milk secreted from hNAG-1 dams has relatively high (~25ng/mL) hNAG-1 protein as well as reduced NEFA concentrations. The presence of hNAG-1 in the mammary gland impaired lactation, contributing to altered perinatal development and lower pup survival. While our study found hNAG-1 concentrations in the milk of hNAG-1 mice, whether this secreted protein is present in human milk remains unknown. Based on our experimental findings related to the impaired lactation observed, it remains to be determine if increased NAG-1 expression is associated with clinical lactational problems in some post-delivery patients or nurturing of the newborn. Current studies suggest the use of NSAIDs are safe while breast-feeding [41], and one study suggests that milk banks could accept donor milk if ibuprofen was taken by the donor [42]. In cells in culture, *in vitro*, some NSAIDs can induce expression of NAG-1, however the data confirming that NSAID increase circulating level *in vivo* in human or experimental animals is not inclusive [25]. Future studies to examine if use of NSAIDs increases circulating NAG-1 levels in the serum and secreted milk of lactating women would be informative. In addition investigation into how NAG-1 alters the expression of *Cidea* the co-activator of C/EBPβ, may provide clues into how NAG-1 alters metabolism.

## Supporting Information

S1 Fig Whole mount analysis of Mammary Gland Development. Virgin glands from WT (A) and hNAG-1 (B) mice appear similar in ductal growth, elongation and branching. Mammary gland differentiation at two magnifications is shown for WT (C&D) and hNAG-1 (E-H) dams on lactation day 2. Glands pictured are representative of lactating dams from both WT or hNAG-1 dams. Some hNAG-1 dams had normal mammary gland development (E&F) while others appeared underdeveloped (G&H).
(TIFF)

S2 Fig Altered cholesterol concentrations in females pup serum during lactation. CD-1 pups were cross-fostered with WT or hNAG-1 dams on PND2 so the pups are all WT CD-1 pups. Pup serum was examined for cholesterol concentrations at PND9 (A&B) or PND21 (C&D). Data shown is average +/- SEM. Mann-Whitney statistical test was done to compare each line to WT control. *, p<0.05; **, p<0.01.
(TIFF)

S3 Fig Non-esterified Fatty Acid concentrations are unaltered in pups nursing from WT or hNAG-1 dams. CD-1 pups were cross-fostered with WT or hNAG-1 dams on PND2 so the pups are all WT CD-1 pups. Pup serum was examined for NEFA concentrations at PND9 (A&B) or PND21 (C&D). Data shown is average +/- SEM.
(TIFF)

S1 Table Genes differentially expressed in hNAG-1 mammary gland. (XLSX)

S2 Table Genes with increased expression in hNAG-1 mammary gland. (XLSX)

S3 Table Genes with decreased expression in hNAG-1 mammary gland. (XLSX)
