# Correction: Expression of Human NSAID Activated Gene 1 in Mice Leads to Altered Mammary Gland Differentiation and Impaired Lactation

**DOI:** 10.1371/journal.pone.0151504

**Published:** 2016-03-10

**Authors:** April K. Binder, Justin P. Kosak, Kyathanahalli S. Janardhan, Glenda Moser, Thomas E. Eling, Kenneth S. Korach

The third author’s name is spelled incorrectly. The correct name is: Kyathanahalli S. Janardhan. The correct citation is: Binder AK, Kosak JP, Janardhan KS, Moser G, Eling TE, Korach KS (2016) Expression of Human NSAID Activated Gene 1 in Mice Leads to Altered Mammary Gland Differentiation and Impaired Lactation. PLoS ONE 11(1): e0146518. doi:10.1371/journal.pone.0146518.
