# Supplementary figures and images for: Expression of Human NSAID Activated Gene 1 in Mice Leads to Altered Mammary Gland Differentiation and Impaired Lactation
Source: PLoS One. 2016 Jan 8;11(1):e0146518. doi: 10.1371/journal.pone.0146518 (PMC4706436; doi:10.1371/journal.pone.0146518)

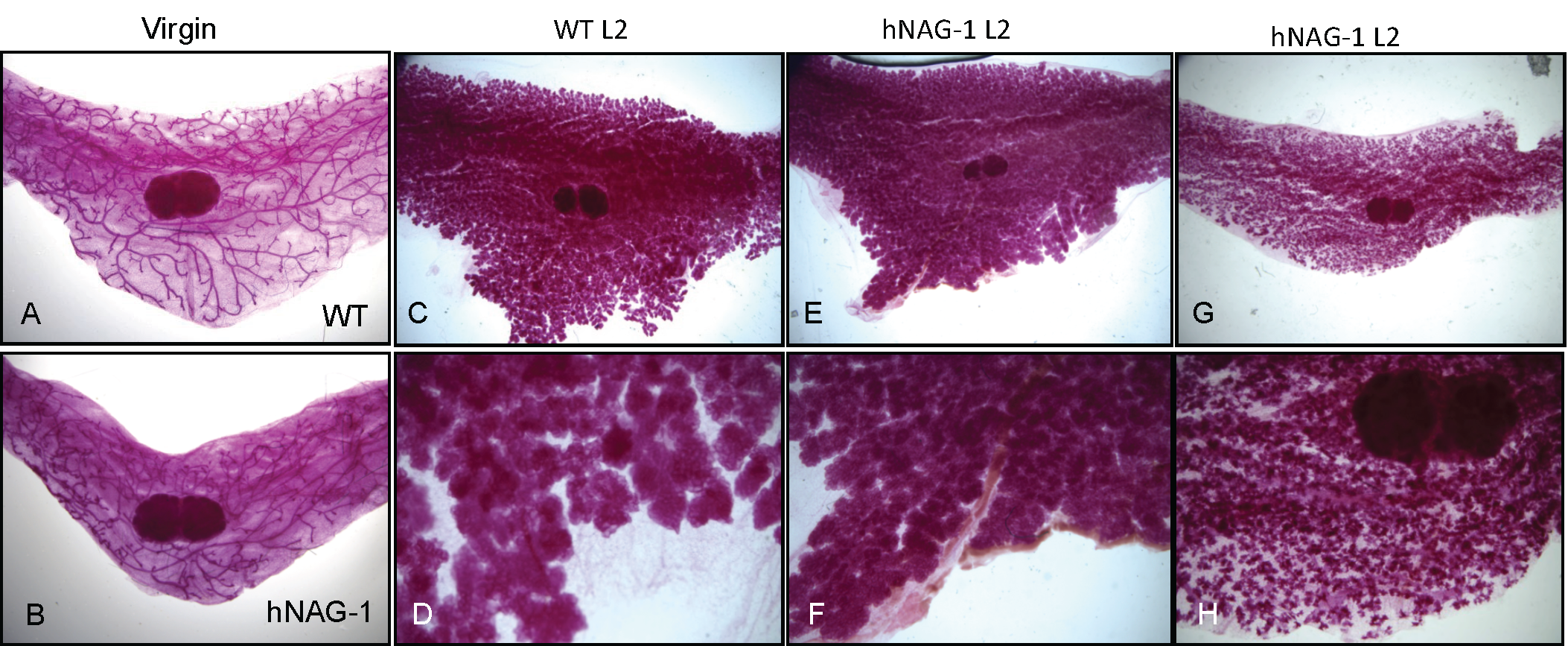

Supplement: S1 Fig — Virgin glands from WT (A) and hNAG-1 (B) mice appear similar in ductal growth, elongation and branching. Mammary gland differentiation at two magnifications is shown for WT (C&D) and hNAG-1 (E-H) dams on lactation day 2. Glands pictured are representative of lactating dams from both WT or hNAG-1 dams. Some hNAG-1 dams had normal mammary gland development (E&F) while others appeared underdeveloped (G&H). (TIFF) [file pone.0146518.s001.tiff]

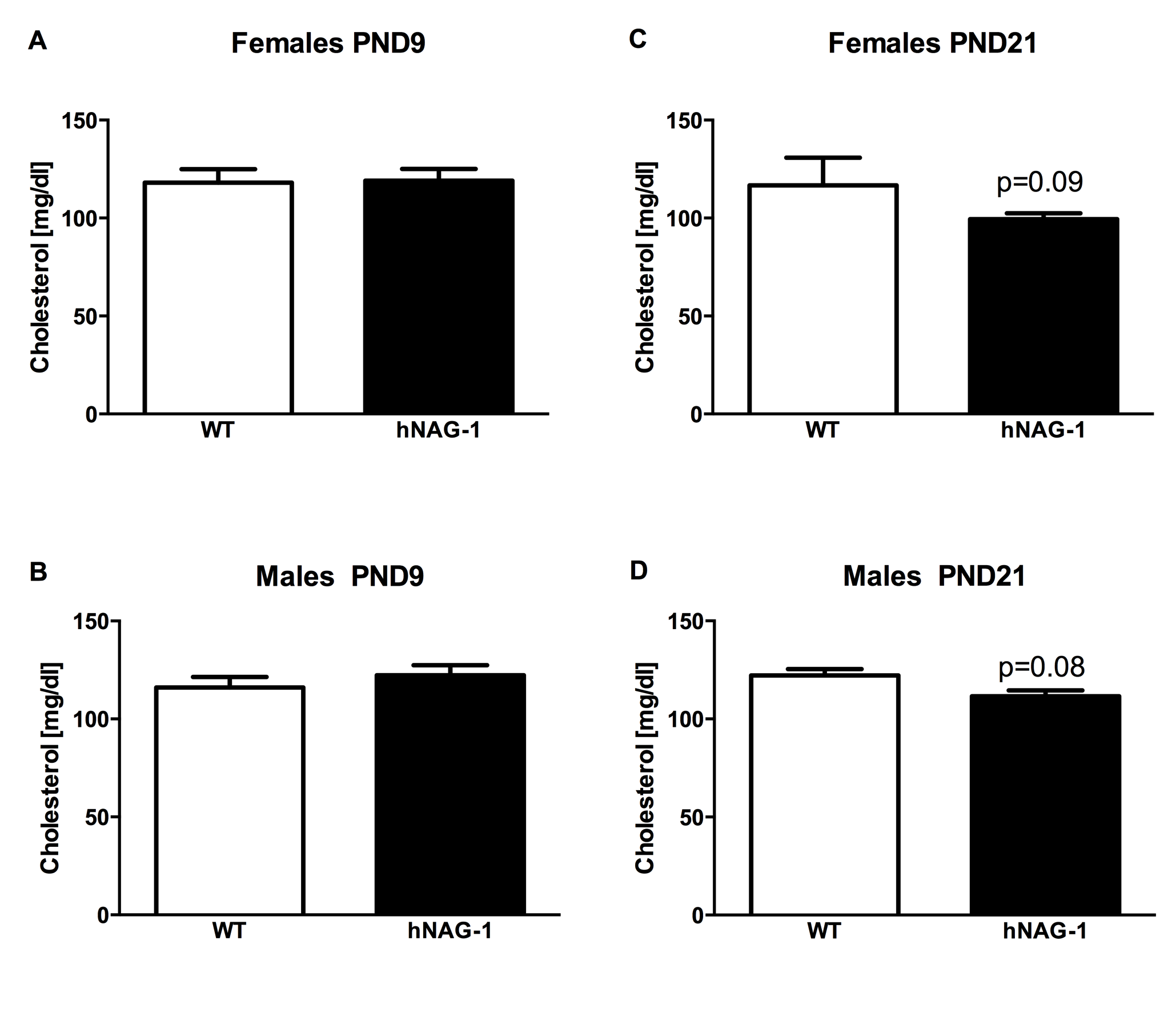

Supplement: S2 Fig — CD-1 pups were cross-fostered with WT or hNAG-1 dams on PND2 so the pups are all WT CD-1 pups. Pup serum was examined for cholesterol concentrations at PND9 (A&B) or PND21 (C&D). Data shown is average +/- SEM. Mann-Whitney statistical test was done to compare each line to WT control. *, p<0.05; **, p<0.01. (TIFF) [file pone.0146518.s002.tiff]

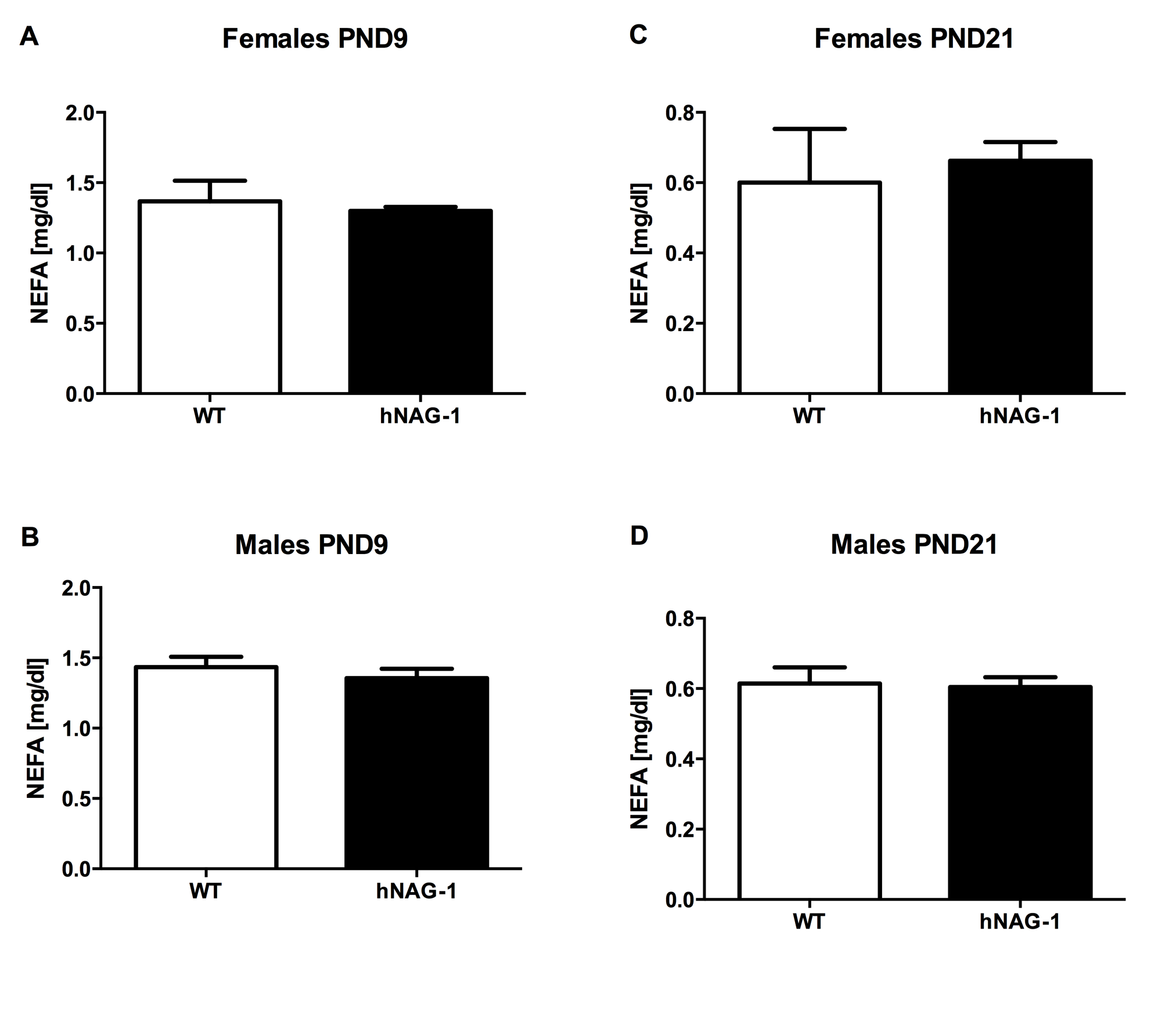

Supplement: S3 Fig — CD-1 pups were cross-fostered with WT or hNAG-1 dams on PND2 so the pups are all WT CD-1 pups. Pup serum was examined for NEFA concentrations at PND9 (A&B) or PND21 (C&D). Data shown is average +/- SEM. (TIFF) [file pone.0146518.s003.tiff]
